# AMIGO3 Is an NgR1/p75 Co-Receptor Signalling Axon Growth Inhibition in the Acute Phase of Adult Central Nervous System Injury

**DOI:** 10.1371/journal.pone.0061878

**Published:** 2013-04-16

**Authors:** Zubair Ahmed, Michael R. Douglas, Gabrielle John, Martin Berry, Ann Logan

**Affiliations:** 1 Neuropharmacology and Neurobiology Section, School of Clinical and Experimental Medicine, University of Birmingham, Birmingham, United Kingdom; 2 Department of Neurology, Dudley Group of Hospitals National Health Service Foundation Trust, Russells Hall Hospital, Dudley, United Kingdom; CNRS UMR7275, France

## Abstract

Axon regeneration in the injured adult CNS is reportedly inhibited by myelin-derived inhibitory molecules, after binding to a receptor complex comprised of the Nogo-66 receptor (NgR1) and two transmembrane co-receptors p75/TROY and LINGO-1. However, the post-injury expression pattern for LINGO-1 is inconsistent with its proposed function. We demonstrated that AMIGO3 levels were significantly higher acutely than those of LINGO-1 in dorsal column lesions and reduced in models of dorsal root ganglion neuron (DRGN) axon regeneration. Similarly, AMIGO3 levels were raised in the retina immediately after optic nerve crush, whilst levels were suppressed in regenerating optic nerves, induced by intravitreal peripheral nerve implantation. AMIGO3 interacted functionally with NgR1-p75/TROY in non-neuronal cells and in brain lysates, mediating RhoA activation in response to CNS myelin. Knockdown of AMIGO3 in myelin-inhibited adult primary DRG and retinal cultures promoted disinhibited neurite growth when cells were stimulated with appropriate neurotrophic factors. These findings demonstrate that AMIGO3 substitutes for LINGO-1 in the NgR1-p75/TROY inhibitory signalling complex and suggests that the NgR1-p75/TROY-AMIGO3 receptor complex mediates myelin-induced inhibition of axon growth acutely in the CNS. Thus, antagonizing AMIGO3 rather than LINGO-1 immediately after CNS injury is likely to be a more effective therapeutic strategy for promoting CNS axon regeneration when combined with neurotrophic factor administration.

## Introduction

CNS axon regeneration is limited by a low intrinsic growth capacity of mature neurons and the presence of a non-permissive environment in the injured adult CNS that inhibits axon growth [1], [2], [3], [4]. Three major inhibitory ligands exist in CNS myelin including Nogo-A, myelin associated glycoprotein (MAG) and oligodendrocyte myelin glycoprotein (OMgp), which collectively account for the majority of CNS inhibitory activity [1], [2], [5], [6]. All three myelin inhibitors bind to a common receptor, Nogo-66 receptor (NgR1) that can signal inhibition and growth cone collapse through the RhoGTPase pathway by associating with two signal transducing binding partners, p75 (the low affinity neurotrophin receptor) and LINGO-1 (leucine rich-repeat and immunoglobulin domain-containing, Nogo receptor interacting protein) [1], [3], [7], [8], [9], [10]. While NgR1 and LINGO-1 are widely expressed in CNS neurons [9], [10], p75 expression is more restricted. TROY, a TNF receptor family member that has a broad pattern of expression in postnatal and adult neurons, was identified as a substitute for p75 in the NgR1/p75/LINGO-1 receptor complex [11], [12].

Other proteins that contain LINGO-1-like LRR motifs include amphoterin (also known as HMGB1), a heparin-binding LRR protein abundant in growth cones [13] and amphoterin-induced gene and open reading frame 1 (AMIGO), along with AMIGO2 and AMIGO3 isoforms, that were isolated from rat brain and shown to have neurite outgrowth promoting properties [14]. AMIGO, AMIGO2 and AMIGO3 are expressed in brain tissues in adult mice, with AMIGO3 having a more widespread distribution also being found in liver, kidney and spleen [14]. Expression of AMIGO correlates with the onset of CNS myelination during postnatal development and localises to axonal fibre tracts, while a substrate bound AMIGO-immunoglobulin fusion protein which antagonizes AMIGO, promotes neurite outgrowth of hippocampal neurons [14], but little is known about the axogenic properties of AMIGO2 and AMIGO3.

Since LINGO-1 expression levels do not rise in the spinal cord until 14 days after spinal cord injury [10], other NgR1 co-receptors mediating axon growth inhibition are likely to be expressed and function during the acute stages after CNS injury. Here, we report using retinal ganglion cell (RGC) and dorsal root ganglion neuron (DRGN) axotomy models that: (1), AMIGO3 mRNA and protein levels are preferentially and significantly raised in DRGN and RGC immediately after central axotomy; (2), depression of AMIGO3 expression correlates with dorsal column (DC) and optic nerve regeneration; (3), AMIGO3 interacts with NgR1 and p75/TROY in both transfected cells and rat and human brain lysates, forming a functional receptor complex that activates RhoGTP in cells exposed to CNS myelin extracts (CME); and (4), siRNA-mediated knockdown of AMIGO3 significantly enhances DRGN and RGC neurite outgrowth in CME-inhibited cultures when stimulated with appropriate neurotrophic factors (NTF). These results suggest that AMIGO3 substitutes for LINGO-1 in centrally axotomized DRGN and RGC in the acute phase of injury and that the NgR1-p75/TROY-AMIGO3 receptor complex mediates immediate axon growth inhibitory responses to CNS myelin.

## Materials and Methods

### Ethics statement

This study was carried out in strict accordance to the UK Animals Scientific Procedures Act, 1986 and all procedures were licensed and approved by the UK Home Office and by the University of Birmingham Ethical Review Sub-Committee. All surgery was performed under inhalation anaesthesia using 5% Isofluorane (IsoFlo, Abbott Animal Health, North Chicago, IL, USA) for induction and 2% for maintenance. Animals were kept in environmentally controlled designated and licenced animal facility at the University of Birmingham and every effort was made to minimise animal suffering throughout the study.

### Regenerating and non-regenerating DRGN models

Regenerating and non-regenerating models were established as described by us [15], [16], [17], [18]. Briefly, experiments comprised 4 groups, each containing 10 adult female Sprague-Dawley rats (180–220 g) (Charles River, Margate, UK) for each analytical time-point and designated as: (1), uninjured control (intact); (2), DC crush (non-regenerating DC model); (3), sciatic nerve (SN) crush (regenerating SN model); and (4), preconditioning (p)SN lesions 1 week before a DC crush (regenerating pSN+DC model). Under inhalation anaesthesia, DC were crushed bilaterally at the level of T8 using calibrated watchmaker's forceps inserted through the dorsal cord meninges to a depth of 1.5 mm. For SN injury, the left SN was exposed at a mid-thigh level and crushed using needle forceps at the level of the sacrotuberous ligament. One week before a DC crush, SN lesions were performed as described above. Animals were killed by CO_2_ exposure at 1 and 10 days post-lesion (dpl) and the L4/L5 DRG harvested for RNA and protein analysis by snap freezing in liquid nitrogen. For immunohistochemistry, animals were intracardially perfused with 4% formaldehyde and the L4/L5 DRG were processed as described later.

### Regenerating and non-regenerating optic nerve (ON) injury models

Regenerating and non-regenerating ON injury models, each containing 12 animals, were established as described by us [19], [20], [21], [22]. Briefly, the ON of adult female 200–250 g Fischer rats were crushed intraorbitally under inhalation anaesthesia, using fine watchmakers forceps. The regenerating ON model (ON-RM) group comprised animals that received intravitreal implantation of 5 mm of freshly teased SN, held in place using Sterispon gelatine sponge (Johnson and Johnson, New Jersey USA) while the non-regenerating model (ON-NRM) received no treatment after ON crush. The changes in AMIGO3 levels after these two treatments were compared to intact controls. Sham injured controls were also included, but these did not demonstrate changes in AMIGO3 levels and therefore are not shown.

### Microarray analysis

The rat genome AROS™ V3.0set (Operon Biotechnologies GmbH, Cologne, Germany) containing 26,962 long mer probes representing 22,012 genes and 27,044 gene transcripts was used for the microarray analysis as described by us previously [16], [17], [23]. Briefly, either Cy3 or Cy5-labelled oligonucleotide probes were hybridised according to the manufacturer's protocol and slides were scanned with an Axon GenePix 4000B scanner (Molecular Devices Ltd, Berkshire, UK). After subtracting background fluorescence values for Cy3 or Cy5 channels in GenePix, data was exported into GeneSpring GX7 (Agilent, Berkshire, UK) and Lowess normalisation carried out. Data was then filtered below P<0.05 threshold and fold changes of greater than 2 were deemed significant. Each condition was replicated at least ×4 in duplicates.

### Semi-quantitative PCR

RNA was extracted from DRG tissues using an RNeasy kit (Qiagen Ltd, Crawley, UK) [17]. Each 25 ng of total RNA sample was analysed on a 1% agarose gel at 3 cycle intervals to ensure exponential PCR product amplification using the following primers: GAPDH 5′-AATGCATCCTGCACCACCAA-3′ and 5′-GTAGCCATATTCATTGTCATA-3′; AMIGO3 5′-CGGCTGCGTGCCTTGTACCT-3′ and 5′-AGCACTTAGGCCCCGCTGGT-3′; p75 5′-CCATCTTGGCTGCTGTGGTT-3′ and 5′-GCTGTTCCATCTCTTGAAAGCAA-3′; NgR1 5′-AAGTGCTGCCAGCCAGATG-3′ and 5′-GCCTCCCGGGTTCCAGTA-3′; TROY/TAJ 5′-CCCTCAATCCCGAAAACGA-3′ and 5′-TGGCCGCCACTGGAAT-3′; LINGO-1 5′-CTTTCCCCTTCGACATCAAGAC-3′ and 5′-CAGCAGCACCAGGCAGAA-3′ (All synthesized by Alta Bioscience, University of Birmingham, UK).

### Immunohistochemistry

Perfusion-fixed L4/L5 DRG were cryoprotected through a graded series of sucrose solutions and blocked in OCT (TAAB Laboratories, Peterborough, UK). Cryosections of DRG were cut 10 µm thick, adhered onto charged glass slides and immunohistochemistry performed as described by us previously [15], [17]. Briefly, sections were washed, permeabilized in PBS containing 1% Triton X-100 (Sigma, Poole, UK) and blocked in PBS containing 0.5% BSA (Sigma) and 0.05% Tween 20, followed by incubation with the relevant antibody in a humidified chamber overnight at 4°C. Primary antibodies included: goat anti-human AMIGO, AMIGO2, AMIGO3 and TROY (all used at 1∶100 dilution and from Santa Cruz Biotechnology, Santa Cruz, CA, USA); monoclonal anti-NGFR (1∶200 dilution; Sigma); rabbit anti-NGFR1 (1∶200 dilution; Alpha Diagnostics, San Antonio, TX, USA); and rabbit anti-LINGO1 (1∶400 dilution; Abcam, Cambridge, UK). Sections were washed in PBS and incubated with relevant secondary antibodies, diluted at 1∶400 and coupled to either Alexa Fluor 488 (green) or Texas Red (red) (both from Invitrogen, Paisley, UK). Controls were included in each run for each antibody that included omission of primary antibody and, where available, pre-incubation with relevant blocking peptides [15], [17], [24]. All controls showed no positive immunoreactivity. Each immunohistochemistry run was performed in duplicate and repeated on three separate occasions. Sections were viewed using a Zeiss epi-fluorescent microscope (Carl Zeiss, Hertfordshire, UK) and photomicrographs were captured using Axiovision Software (Zeiss) and a Zeiss HRc camera (Zeiss).

### Cloning of full-length p75, TROY, NgR1 and AMIGO3 and transfection of COS7 cells

Full length rat NgR1 p75, TROY and AMIGO3 (PubMed accession numbers: NM_031831, NM_012610, NM_ DQ466087.1 and NM_178144.1, respectively) gene sequences were PCR amplified and the p75 and TROY genes were cloned as BglII/EcoRI fragments into a pCMV-HA plasmid (Invitrogen), while AMIGO3 gene was cloned into the pCMV-Flag plasmid (Invitrogen). Five cytoplasmic tail truncation mutants of full length AMIGO3 (residues 1–508, transmembrane region 384–404) were created by PCR using a common 5′ forward primer and five appropriate reverse primers (sequences available upon request). This resulted in the following cytoplasmically truncated (CT) constructs: AMIGO3 CT1 (1–409), CT2 (1–424), CT3 (1–444), CT4 (1–464) and CT5 (1–484). Transfection of these constructs into COS7 cells showed that AMIGO3 CT1 (cytoplasmic region of 5 amino acids) was sufficient for efficient cell surface expression and, for all experiments described in this manuscript, AMIGO3 CT1 refers to this construct. Expression constructs for each gene were sequenced to confirm the correct sequence and reading frame. COS7 cells were maintained in DMEM supplemented with 10% foetal calf serum and grown at 37°C in a humidified atmosphere containing 5% CO_2_. To express target cDNA in COS7 cells, 2×10^6^ cells were plated onto 100 mm dishes and 10 µg of pCMV-HA-p75, pCMV-Flag-AMIGO3, pCMV-Flag-AMIGO3 CT1 and pCMV-NgR1 were co-transfected in different combinations using Lipofectamine 2000, following the manufacturer's guidelines (Invitrogen).

### Immunoprecipitation and western blots

COS7 cells, transfected with combinations of Flag-AMIGO3, NgR1 (untagged), p75-HA or TROY-HA, were harvested after 48 h and lysed in 1 ml ice-cold lysis buffer (50 mM HEPES (pH 7.5), 150 mM NaCl, 1.5 mM MgCl2, 1 mM EGTA, 1% NP-40 and 10% glycerol) for 30 min at 4°C. Cell lysates were then cleared by centrifugation at 13,000×g for 15 min at 4°C. Supernatants were incubated with Protein A/G-Sepharose beads (Sigma) at 4°C for 1 hr and then incubated with either anti-Flag M2 affinity gel (1∶500 dilution; Sigma), anti-HA affinity matrix (1∶500 dilution; Roche, West Sussex, UK) or affinity purified rabbit anti-NgR1 antibody (1∶500 dilution; Alpha Diagnostics). Beads were then washed ×3 with lysis buffer, boiled in 2× Laemmli sample buffer for 4 min, subjected to 10% SDS-PAGE, and analysed by western blotting with the following antibodies: anti-Flag M2 (1∶500 dilution; Sigma), anti-HA (1∶500 dilution; Roche) and anti-NgR1 (1∶500 dilution; Alpha Diagnostics). Bands were visualised with appropriate HRP-labelled anti-rabbit or anti-mouse IgG (1∶1000 dilution; GE Healthcare, Buckinghamshire, UK) and the signal was developed using an ECL chemiluminescence kit following the manufacturer's instructions (GE Healthcare).

For co-immunoprecipitation of endogenous target proteins from rat (7 week old adult female Sprague-Dawley rat weighing 180 g) and human brain (49 year old female who died from cardiac arrest), 10 mg of each tissue was homogenised in ice-cold lysis buffer (described earlier), centrifuged for 15 min at 13,000×g and supernatants were stored until required. Dynabeads coated with Protein G were then used to immunoprecipitate specific target antigens according to the manufacturer's instructions (Invitrogen). Briefly, either 5 µg of goat anti-human AMIGO3 (1∶500 dilution; Santa Cruz Biotechnology) or rabbit anti-human NgR1 (1∶500 dilution; Alpha Diagnostics) or monoclonal anti-NGFR (1∶1000 dilution; Sigma) antibodies diluted in 200 µl PBS containing 0.1% Tween 20 was incubated with gentle agitation for 10 min at room temperature before harvesting the Dynabeads coated with relevant antibodies (Dynabeads-Ab complex) by placing tubes in a DynaMag-2 (Invitrogen). The bead supernatant was removed and replaced with 750 µl of each lysate from brain tissue lysates and incubated for 30 min at room temperature with gentle agitation. Tubes were placed in the DynaMag-2 to collect the Dynabeads-Ab-antigen complexes which were washed ×3 in PBS and boiled in 2× Laemmli buffer for 4 min. Tubes were then placed on the DynaMag-2 to collect the beads, supernatants containing the proteins of interest were removed, resolved on 10% SDS-PAGE gels and analysed by western blotting with relevant antibodies (same dilutions as above) to AMIGO3, p75 and NgR1 as detailed above.

### Densitometry

Western blots were scanned into Adobe Photoshop (Adobe Systems Inc, San Jose, CA, USA) keeping all scanning parameters the same between blots. Bands were then analysed using the built-in-macros for gel analysis in ImageJ (NIH, USA, http://imagej.nih.gov/ij), normalized to GAPDH or β-actin, where appropriate, and means ± SEM were plotted in Microsoft Excel (Microsoft Corporation, CA, USA) [17], [25], [26], [27].

### Rho activation assay

COS7 cells were transfected with combinations of Flag-AMIGO3, Flag-AMIGO3 CT1, NgR1 (untagged), HA-p75, HA-TROY, as described previously. After 48 h, the transfected cells were washed ×3 in PBS and exposed to 10 µg/ml adult Sprague-Dawley rat CNS myelin extracts (CME) (prepared as described in [25]) for 15 min. Cells were then washed ×3 in PBS and lysed with ice-cold lysis buffer (50 mM Tris-HCl (pH 7.5), 1% Triton X-100, 0.5% sodium deoxycholate, 0.1% SDS, 500 mM NaCl, 10 mM MgCl2 and 5 µg/ml protease inhibitor cocktail (Sigma)). GTP bound RhoA was assayed using a Rho pulldown assay (Millipore, Watford, UK) and/or a colorimetric G-Lisa Rho activation assay kit (Cytoskeleton Inc, Denver, CO, USA) following the manufacturer's instructions.

### Adult rat DRG cell cultures

Dissociated mixed primary adult rat DRG cell cultures were prepared from L4-L7 DRG pairs as described by us previously [16], [17]. Briefly, DRG cells were dissociated using collagenase (Sigma), triturated to break up the digested tissues and cell debris removed by centrifugation through a 15% bovine serum albumin (Sigma) gradient. 500 DRGN/well were cultured on sterile glass 8-well chamber slides (BD Biosciences, Oxford, UK) pre-coated with poly-D-lysine and laminin-I (both from Sigma) either in the presence or absence of 100 µg/ml CME [25] for at least 72 h. 5-fluoro-2-deoxyuridine (5-FDU) (Sigma) was added immediately after plating cells at a final concentration of 30 µM to block glial cell proliferation [25]. Fibroblast growth factor-2 (FGF2 from Peprotech, London, UK) was used at a final concentration of 10 ng/ml to drive neurite outgrowth for 72 h [15], [17], [25], [26].

### Adult rat retinal cultures

Retina cultures containing RGC were dissociated from adult rat retina (6–8 weeks old) using a papain system (Worthington Biochemicals, New Jersey, USA), as described by us [22], [24] and 125×10^3^ dissociated cells were plated in 8-well chamber slides (Beckton Dickinson) in supplemented Neurobasal-A (Invitrogen) either in the presence or absence of CME (10 µg/ml) and CNTF (10 ng/ml; Peprotech) with 30 µM 5-FDU for 72 h.

### Immunocytochemistry

PBS washed DRG/retinal cultures were fixed in 4% formaldehyde, washed in PBS, blocked in PBS containing 0.5% BSA and 0.1% Tween 20, and incubated with the relevant antibody for 1 h at room temperature in a humidified chamber, as described by us previously [16]. Briefly, DRG/retinal cells were either double stained for AMIGO3 (1∶200 dilution; Santa Cruz) and βIII-tubulin (1∶200; Sigma) or βIII-tubulin alone (1∶200 dilution; Sigma) to detect neurite outgrowth for 1 h at room temperature. After washing ×3 in PBS, cells were incubated with either Alexa 488 or Texas Red-labelled relevant secondary antibodies. Cells were then washed ×3 in PBS and mounted in Vectamount containing DAPI (Vector Laboratories).

### AMIGO3 siRNA and transfection

An siRNA sequence directed against AMIGO3 (siAMIGO3, sense 5′-TGGAGGAGCTGGAGAAGTT-3′) was purchased from Dharmacon (Colorado, USA) and 50 nM of siAMIGO3 was used to knock down AMIGO3 mRNA in DRGN/RGC in mixed cultures, as described by us previously [15], [17], [25], [28]. DRGN/RGC were also transfected with Lipofectamine alone (control), a scrambled version of the above siAMIGO3 sequence (scr-siAMIGO3; Dharmacon) and a non-specific siRNA targeting green fluorescent protein (siGFP; Dharmacon) as controls. Cultures were transfected for 5 hours prior to replacement of the transfection medium with fresh Neurobasal-A with or without CME/with or without FGF2/CNTF, as appropriate. After 72 h, cultures were either fixed in 4% paraformaldehyde for immunocytochemistry or lysed in ice-cold cell lysis buffer for western blot or RhoGTP analyses. Each experiment was performed in triplicate and repeated on 3 separate occasions. Western blot and subsequent densitometry was used to quantify the extent of AMIGO3 knockdown, as described above and by us previously [17], [24], [25], [28].

### Measurement of neurite outgrowth and percentage of DRGN/RGC with neurites

To measure neurite outgrowth, each well of an 8-well chamber slide was divided into 9 equal quadrants and photographs were captured randomly from these quadrants using Axiovision (Carl Zeiss, Hertfordshire, UK). At least 30 βIII-tubulin^+^ DRGN/well and all RGC with neurites were analysed to measure the longest neurite for each DRGN/RGC using the built-in measurement facilities in Axiovision software (Carl Zeiss), as described by us previously [15], [17], [25]. Each data point, therefore, represents measurements of at least 180 DRGN or RGC/condition.

To count the percentage of DRGN/RGC with neurites, chamber slides were anonymised by a second investigator and the number of DRGN/RGC with neurites were using a Zeiss epifluorescent microscope (Carl Zeiss) scanning the entire well of each 8-well chamber for each condition (n = 6 wells/condition). Neurites were defined as βIII-tubulin^+^ processes emanating from the DRGN/RGC cell body with lengths equal to or longer than the DRGN/RGC diameter [25], [26].

### Statistical analysis

Each experiment was repeated on 3 independent occasions and sample means were calculated for each variable and analysed for significance by one-way analysis of variance (ANOVA) followed by post-hoc testing with Dunnett's method.

## Results

### AMIGO3 and not LINGO1 mRNA levels are significantly elevated in DRGN 1 day after DC crush

**Figure 1:**
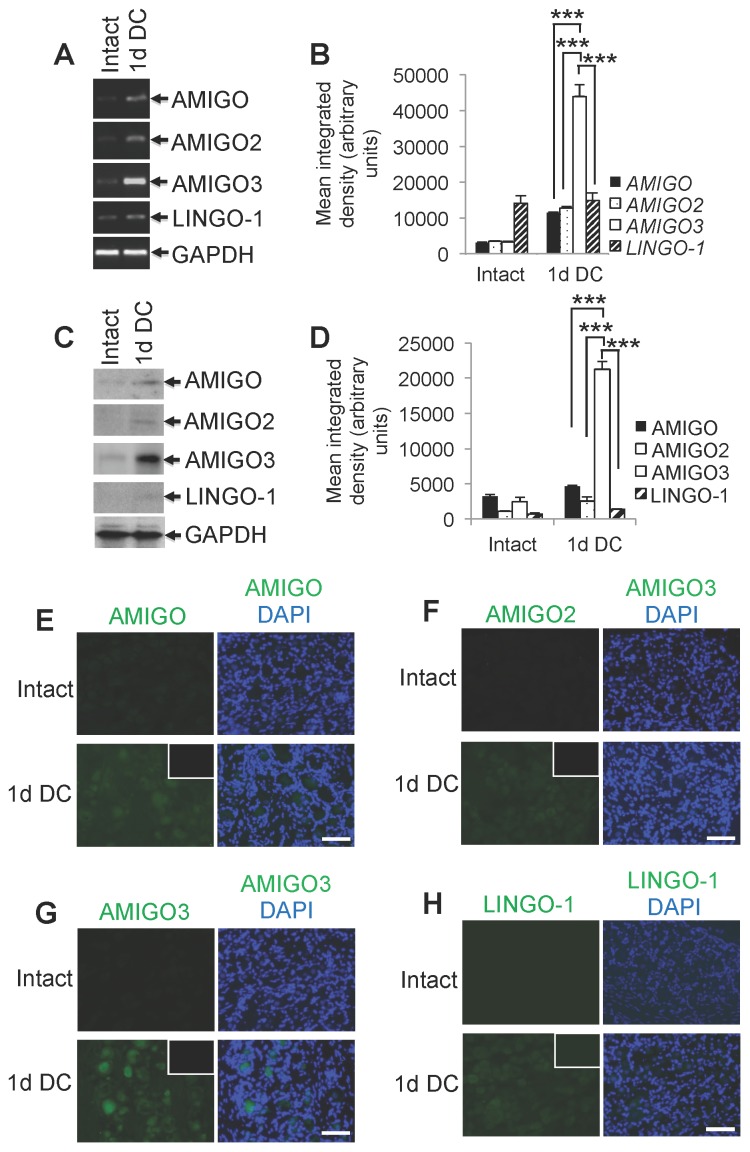
Levels of AMIGO at 1 day after DC injury. (**A**) Semi-quantitative RT-PCR and (**B**) densitometry to show changes in mRNA levels of AMIGO isoforms and LINGO-1 expressed in non-regenerating DRGN compared to intact controls. GAPDH is used as a housekeeping gene. (**C**) Representative western blots and (**D**) densitometry to show changes in protein levels of AMIGO isoforms and LINGO-1 in non-regenerating DRGN relative to intact controls. (**E–H**) Immunohistochemistry in sections from intact controls and 1 d DC injured DRGN shows immunolabelling for AMIGO (**E**), AMIGO2 (**F**), AMIGO3 (**G**) and (**H**) LINGO-1. Insets in E-H show images from blocking peptide controls to demonstrate specificity of the relevant antibody Scale bars in E–H = 100 µm. *** = P<0.0001, ANOVA.

At 1 day after DC injury, we found by microarray that AMIGO, AMIGO-2 and LINGO-1 mRNA levels were not modulated (**Table 1**), whilst AMIGO3 levels were significantly elevated by 4-fold compared to intact control DRG (**Table 1**). Semi-quantitative RT-PCR (**Figure 1A and B**) and western blot (**Figure 1C and D**) analysis confirmed these changes at 1 day after DC crush, demonstrating significant up-regulation of AMIGO3 mRNA and protein levels (greater than 20-fold) when compared to intact control, AMIGO and AMIGO2 (>4-fold increase), with little or no change in LINGO-1 mRNA or protein levels (**Figure 1A–D**). Immunohistochemistry for AMIGO (**Figure 1E**), AMIGO2 (**Fig. 1F**), AMIGO3 (**Fig. 1G**) and LINGO-1 (**Fig. 1H**) in sections of DRG taken at 1 day after DC crush, mirrored changes observed for mRNA levels and demonstrated that AMIGO3, but not AMIGO proteins were elevated in DRGN. Together, these data indicate that AMIGO3 mRNA and protein levels are significantly up-regulated above those of other AMIGO isoforms and LINGO-1 at 1 day after DC injury.

**Table 1:** Microarray analysis to show fold-differences in mRNA levels compared to intact controls of described genes in rat DRG 1 d after DC lesion.

Gene	Description	1 d post-DC ± SEM
*AMIGO*	Amphoterin induced Gene and ORF	1.01±0.02
*AMIGO2*	Amphoterin induced Gene and ORF2	1.02±0.01
*AMIGO3*	Amphoterin induced Gene and ORF3	4.04±0.04***
*LINGO1*	Leucine Rich Repeat and Ig domain 1	1.03±0.01

Mean values are shown from 4 different samples run in duplicate.

*** P<0.0001.

### At 10 days, AMIGO3 levels are significantly increased in DRGN in the non-regenerating DC lesion model

**Figure 2:**
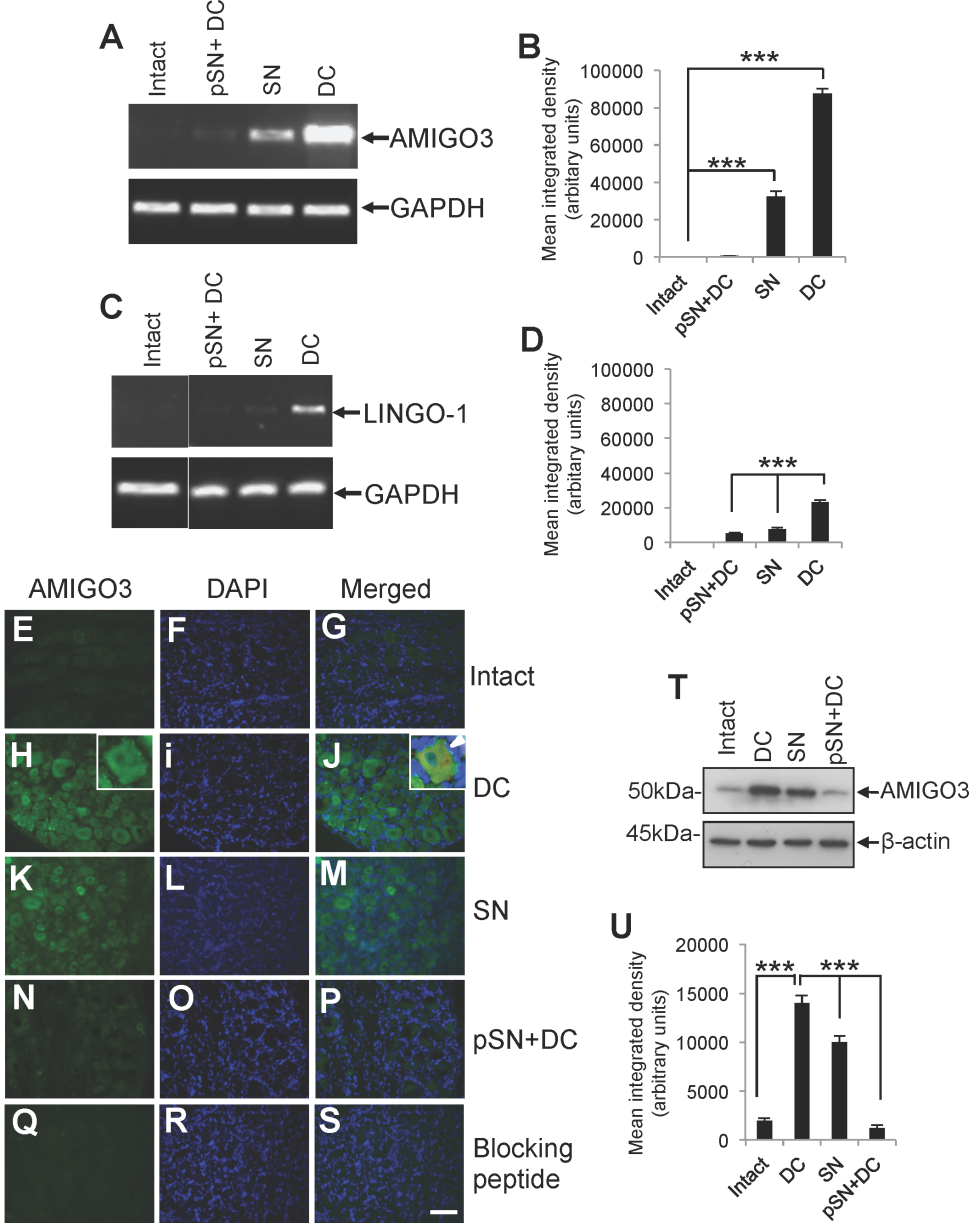
AMIGO3 levels at 10 days in intact controls, non-regenerating DC, and regenerating SN and pSN+DC models. (**A**), (**C**) Semi-quantitative RT-PCR and (**B**), (**D**) densitometry was used to quantify AMIGO3 and LINGO-1 expression levels using total RNA extracted from appropriately treated or untreated DRG. (**E–S**) Immunohistochemistry, (**T**) western blotting and (**U**) densitometry to show high levels of AMIGO3 localisation in DRGN from non-regenerating DC models compared to those in regenerating SN and pSN+DC models. High power insets in (**H**) and (**J**) show neuronal and glial localisation of AMIGO3 in sections double stained with Neurofilament 200 and AMIGO3 (arrowhead). Blocking peptide control (**Q–S**) shows the specificity of AMIGO3 antibodies used. Scale bar = 100 µm. *** = p<0.0001, ANOVA.

Using microarray analysis at 10 days after injury, we found >8-fold increase in DRG AMIGO3 mRNA in the non-regenerating DC model, compared to intact controls (**Table 2**). In the SN paradigm (peripheral nerve regeneration paradigm), DRG AMIGO3 levels were less, at >3.5-fold higher than those of intact controls, while in the regenerating pSN+DC paradigm, AMIGO3 mRNA was unchanged compared to intact controls (**Table 2**). At this later sub-acute time point, DRG LINGO-1 mRNA levels had risen by 2-fold in the non-regenerating DC model compared to those in intact control DRG. All these changes were confirmed by semi-quantitative RT-PCR and subsequent densitometric analysis (**Figure 2A–D**).

**Table 2:** Microarray analysis to show fold-differences in mRNA levels of described genes compared to intact controls in rat DRG 10 d after DC lesion, sciatic nerve (SN) or pre-conditioning (pSN+DC) lesions.

Gene	Description	DC	SN	pSN+DC
*AMIGO*	Amphoterin induced Gene and ORF	1.77±0.01	1.02±0.02	1.03±0.02
*AMIGO2*	Amphoterin induced Gene and ORF2	1.85±0.04	1.01±0.02	1.02±0.05
*AMIGO3*	Amphoterin induced Gene and ORF3	8.30±0.02***	3.61±0.04	1.04±0.03
*LINGO1*	Leucine Rich Repeat and Ig domain 1	2.06±0.02	1.01±0.01	1.05±0.01

Mean ± SEM values are shown from 4 different samples run in duplicate.

*** P<0.0001.

Little or no changes in levels of AMIGO and AMIGO2 protein were detected in DRGN from non-regenerating DC and regenerating SN and pSN+DC models by immunohistochemistry (not shown). Very low levels of AMIGO3 protein were detected in DRGN from intact control DRG (**Figure 2E–G**), while higher levels of AMIGO3 were observed in DRGN from the non-regenerating DC model (**Figure 2H–J**). Double immunohistochemistry for neurofilament-200 and AMIGO3 also showed that AMIGO3 localisation (inset to Figure 2H) was not restricted to DRGN but also present in DRG glia, including satellite cells (inset to Fig. 2J). In the regenerating SN paradigm however, lower levels of AMIGO3^+^ staining were apparent in DRGN compared to those seen in DRGN in non-regenerating DC models (**Figure 2K–M**). By contrast, DRGN in the regenerating pSN+DC model had low levels of AMIGO3 immunostaining, similar to the low levels detected in intact controls (**Figure 2N–P**). A blocking peptide control demonstrated specificity of the AMIGO3 antibodies used in this study (**Figure 2Q–S**). The differences in immunohistochemistry patterns between models were corroborated by western blotting of DRG protein lysates (**Figure 2T**) and densitometric quantification (**Figure 2U**), confirming that at 10 days significantly lower levels of AMIGO3 were present in DRG tissue in regenerating SN and pSN+DC lesioned models compared to the non-regenerating model. Although the SN model represents a regenerating model, DRGN had significantly higher levels of AMIGO3 in this paradigm compared to the regenerating pSN+DC lesion model, suggesting that disinhibition is achieved in the SN model by alternative and currently unknown mechanisms.

**Figure 3:**
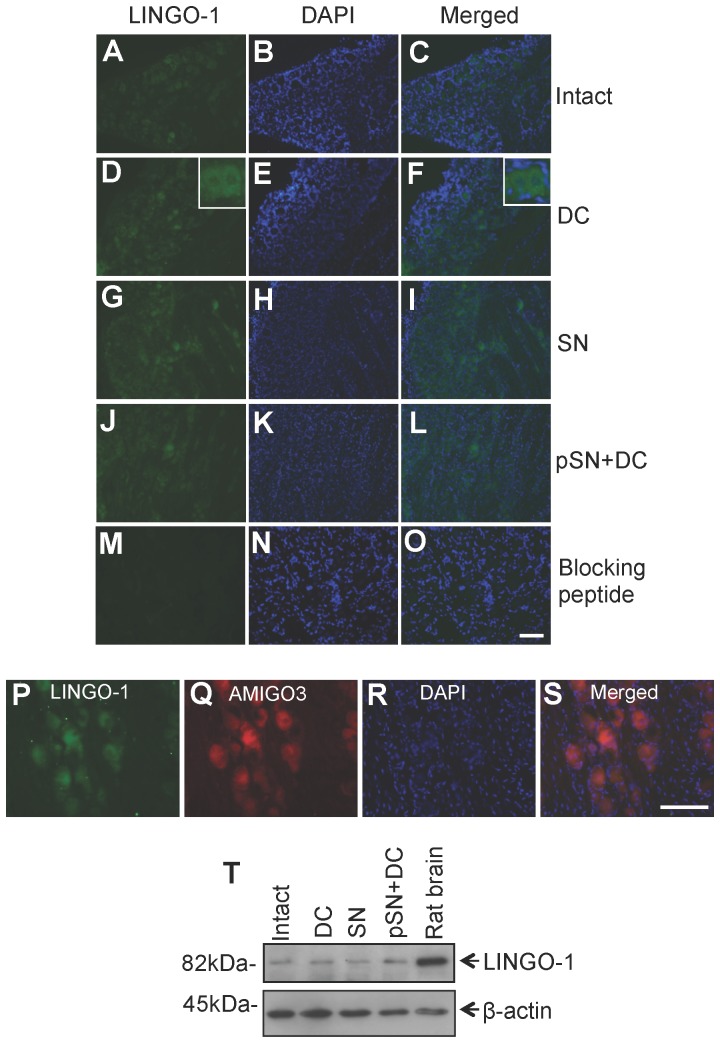
LINGO1 levels in intact, non-regenerating DC and regenerating (SN and pSN+DC lesioned) DRGN models 10 days after injury. High power insets in (D) and (F) show neuronal and glial localisation of AMIGO3. (**P–S**) Co-localisation of LINGO-1 with AMIGO3 in DRGN from non-regenerating DC models. (**T**) Western blotting of LINGO-1 levels in DRG in our experimental model paradigms showing low and unchanged levels of LINGO-1 compared to a positive control lane using rat brain lysates. β-actin was used as a loading control. Scale bars = 100 µm.

Very low levels of LINGO-1 protein were observed by immunohistochemistry at 10 days after injury in both non-regenerating and regenerating DRGN from DC or SN axotomy models, respectively, compared to intact controls (**Figure 3A–O**). Despite LINGO-1 being co-localised with AMIGO3 (**Figure 3P–S**) and LINGO-1 mRNA levels being 2-fold elevated in the non-regenerating DC model, LINGO-1 protein levels did not change and were not correlated with the DRGN regeneration state nor with AMIGO3 expression during the 1–10 day period studied (**Figure 3T**). Thus, AMIGO3 is likely to be more important than LINGO-1 in mediating the inhibition of DRGN axon regeneration at least over the first 10 d after DC axotomy.

### Depressed AMIGO3 levels correlate with RGC axon regeneration

**Figure 4:**
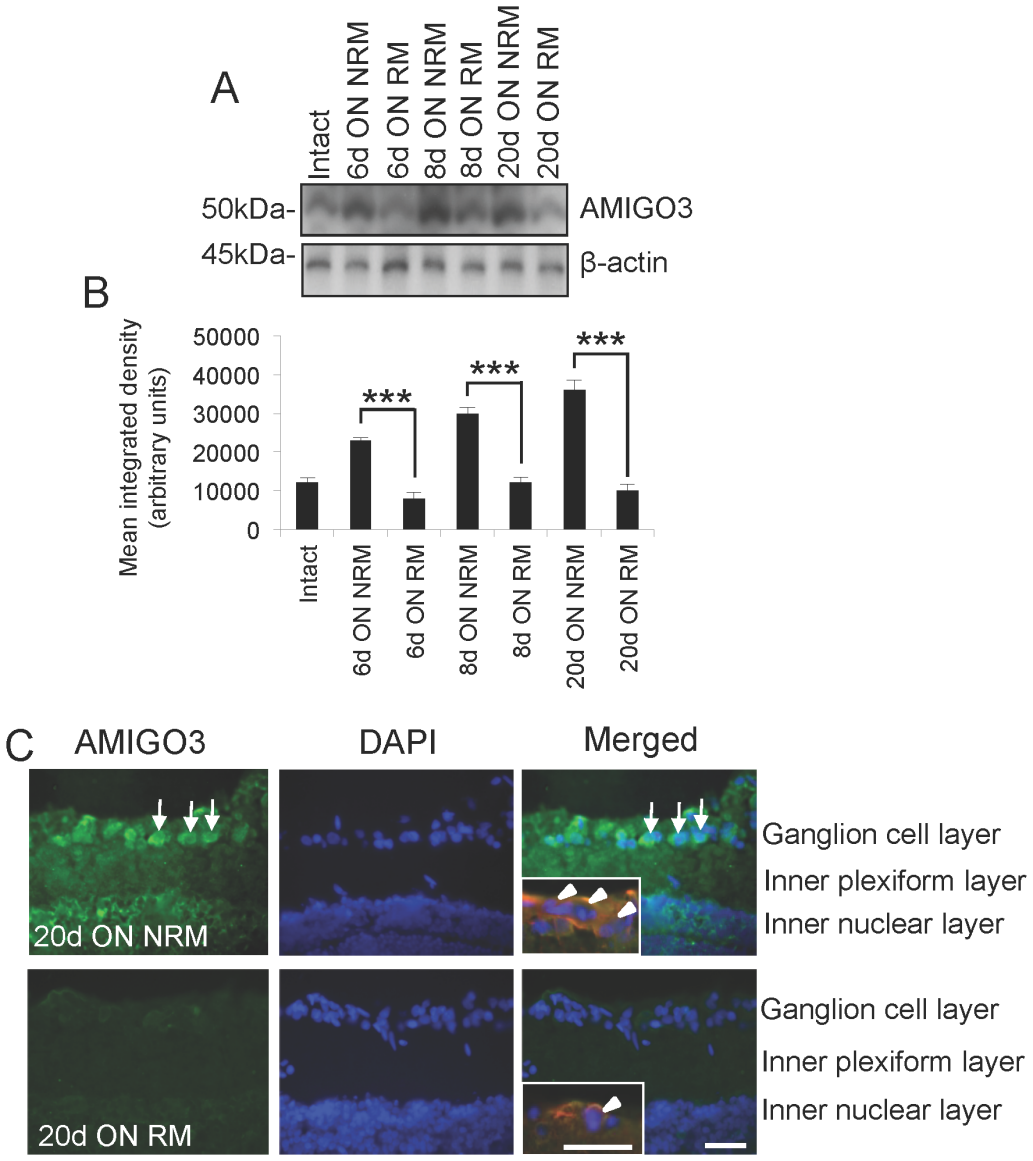
Suppressed levels of AMIGO3 correlate with optic nerve regeneration. (**A**) Representative western blot of AMIGO3 in NRM and RM at 0, 6, 8 and 20 days after ONC and (**B**) densitometry to show higher levels of AMIGO3 in NRM at 6, 8 and 20 days after ONC. β-actin was used as a protein loading control. (**C**) Immunohistochemistry to show that high levels of AMIGO3 is expressed in the majority of RGC in NRM (e.g. arrows) while lower levels are present in RGC from RM retinae. Insets show co-localisation of βIII-tubulin with AMIGO3 (arrowheads). Scale bar in (C) = 100 µm, in insets = 20 µm. *** = P<0.0001, ANOVA.

To confirm our findings in the DRGN injury model, we investigated the dynamic expression levels of AMIGO3 in the ON-RM and ON-NRM and observed that AMIGO3 levels were significantly higher in the ON-NRM and suppressed in the ON-RM at 6, 8 and 20 days after ON crush injury by western blotting and densitometry (**Figure 4A and B**). Immunohistochemistry demonstrated a high frequency of AMIGO3^+^ RGC in the ON-NRM while low levels were detected in a small number of RGC in ON-RM retinae (**Figure 4C**). Double immunolocalisation of AMIGO3 with βIII-tubulin confirmed that AMIGO3 was expressed in βIII-tubulin^+^ RGC (**high power insets in** **Fig. 4C**). These results confirmed our findings in the DC injury model and demonstrated that low levels of AMIGO3 also correlated with RGC axon regeneration the ON injury model.

In light of the *in vivo* observations in the DRGN and ON injury models, we next investigated the possibility that AMIGO3 could substitute for LINGO-1 in the NgR1-p75/TROY inhibitory signalling complex.

### AMIGO3 interacts with NgR1 and p75 in transfected COS7 cells and endogenous rat and human brain lysates

**Figure 5:**
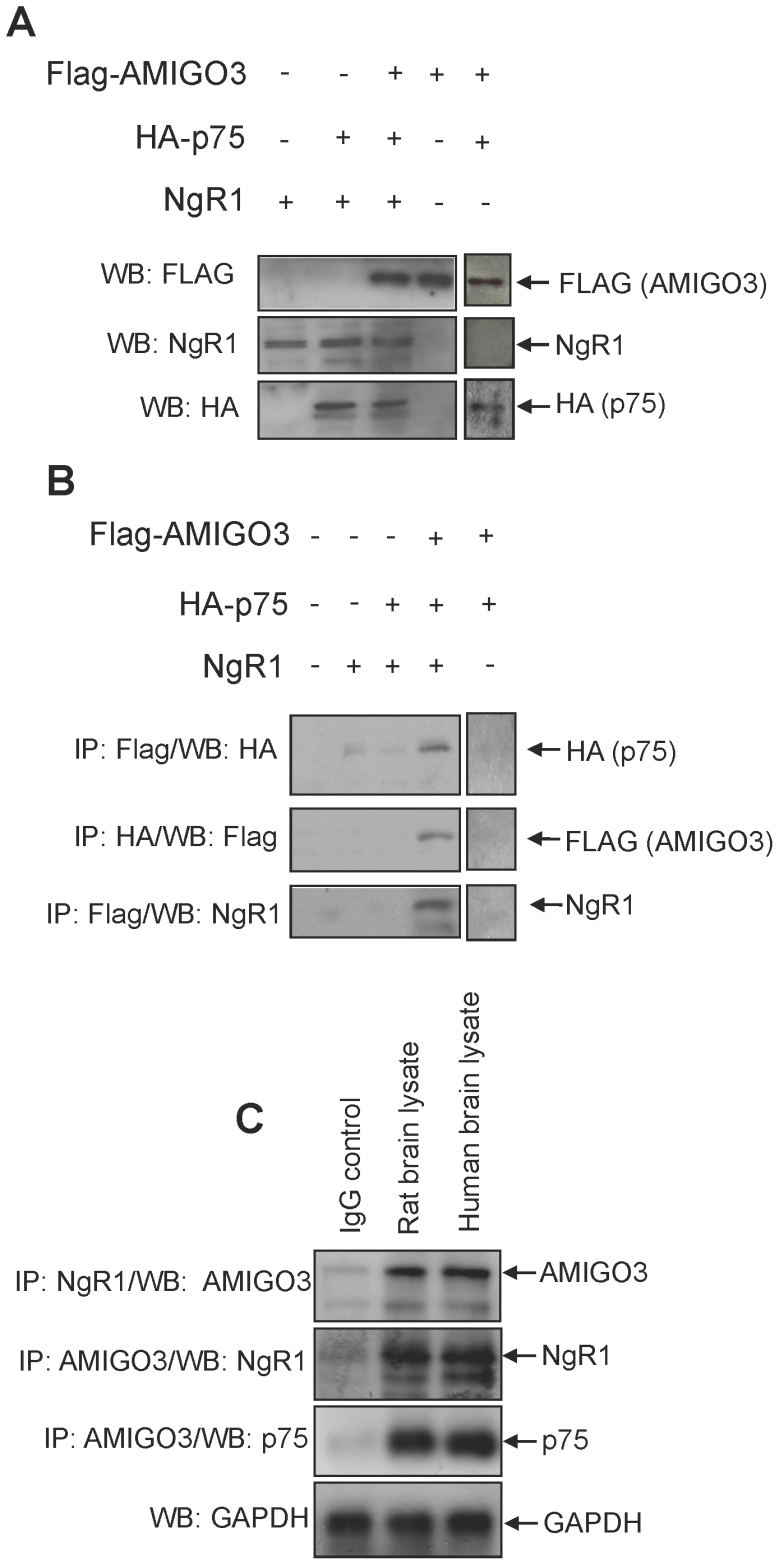
AMIGO3 interacts with NgR1 and p75. (**A**) Western blot of COS-7 total cell lysates extracted 48 h after transfection with combinations of FLAG-AMIGO3, HA-p75 and NgR1 genes. (**B**) Co-immunoprecipitation of AMIGO3, NgR1 and p75 detected after western blotting for relevant anti-HA, anti-FLAG or rabbit anti-NgR1 antibodies from COS-7 cells co-expressing combinations of Flag-AMIGO3, NgR1 and HA-p75. (**C**) Co-immunoprecipitation of AMIGO3, NgR1 and p75 from adult rat and human brain lysates using combinations of goat anti-AMIGO3, rabbit anti-NgR1 and mouse anti-p75 antibodies for immunoprecipitation (IP) and western blotting (WB). IgG was used as a control antibody for IP and GAPDH was used as a loading control.

To determine whether AMIGO3 is a co-receptor for the NgR1-p75 receptor complex, we transfected COS7 cells with combinations of full length Flag-tagged AMIGO3, NgR1 and HA-tagged p75 genes. We confirmed expression of the relevant proteins by western blotting in cell lysates (**Figure 5A**). Co-immunoprecipitation and western blotting for relevant proteins in cells transfected with Flag-AMIGO3, NgR1 and HA-p75 gene combinations demonstrated interaction between AMIGO3, NgR1 and p75 only when all three receptors were co-transfected (**Figure 5B**).

Co-immunoprecipitation, followed by western blotting with relevant antibodies, also showed positive bands and hence binding between NgR1-p75-AMIGO3 protein combinations in both rat and human brain lysates (**Figure 5C**). These results demonstrate that AMIGO3, NgR1 and p75 interact appropriately to form a receptor complex in both transfected cells and in brain extracts containing native co-receptor partners.

### RhoA activation in NgR1-p75/TROY-AMIGO3 transfected COS7 cells

**Figure 6:**
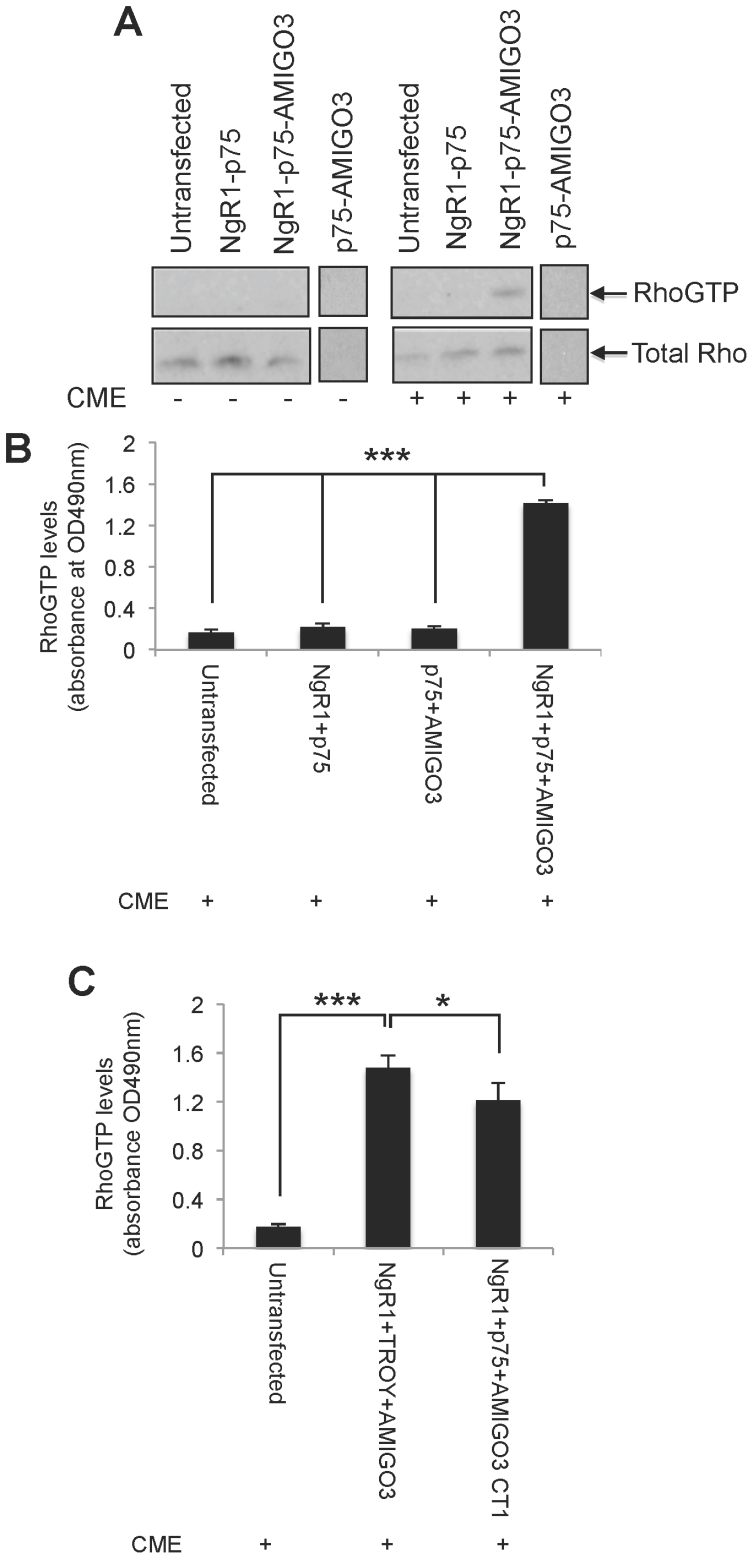
Co-transfection of AMIGO3, NgR1 and p75 activates RhoA. (A) RhoGTP levels detected by a Rho pulldown assay in control untransfected and transfected COS-7 cells expressing NgR1-p75 and NgR1-p75-AMIGO3 genes, in the presence (+) and absence of CME (−). Total Rho levels were also detected by western blotting. (**B**) Colorimetric Rho activation assay in untransfected control and in COS7 cells transfected with NgR1-p75 and NgR1-p75-AMIGO3 gene combinations in the presence of CME (+). (**C**) Colorimetric Rho activation assay in untransfected control COS7 cells and cells transfected with NgR1-TROY-AMIGO3, NgR1-p75-AMIGO3 CT1-^−^ genes, in the presence of CME (+).

In either untransfected COS7 cells, or those transfected with full-length NgR1 and p75 or p75 and AMIGO3 genes together, Rho was not activated either in the presence or absence of CME (**Figure 6A and B**). In the presence of CME, transfection with all three receptors, NgR1, p75 and AMIGO3 genes, conferred responsiveness to CME as evidenced by significantly enhanced RhoGTP levels, detected by both Rho pulldown (**Figure 6A**) and colorimetric RhoGTP assays (**Figure 6B**). Transfection of AMIGO3 along with NgR1 and TROY genes also conferred CME responsiveness similar to that of the NgR1-p75-AMIGO3 genes (**Figure 6C**). Furthermore, transfection with a cytoplasmic tail deleted AMIGO3 (AMIGO3 CT1) with NgR1 and p75 also conferred CME responsiveness of cells. (**Figure 6C**). Our results demonstrate that AMIGO3 forms a functional complex with NgR1 and p75/TROY to confer responsiveness to CME and that only the extracellular portion of AMIGO3 is required for this functional interaction.

### Knockdown of AMIGO3 promotes disinhibited DRGN neurite outgrowth in the presence of CME only when stimulated with FGF-2

**Figure 7:**
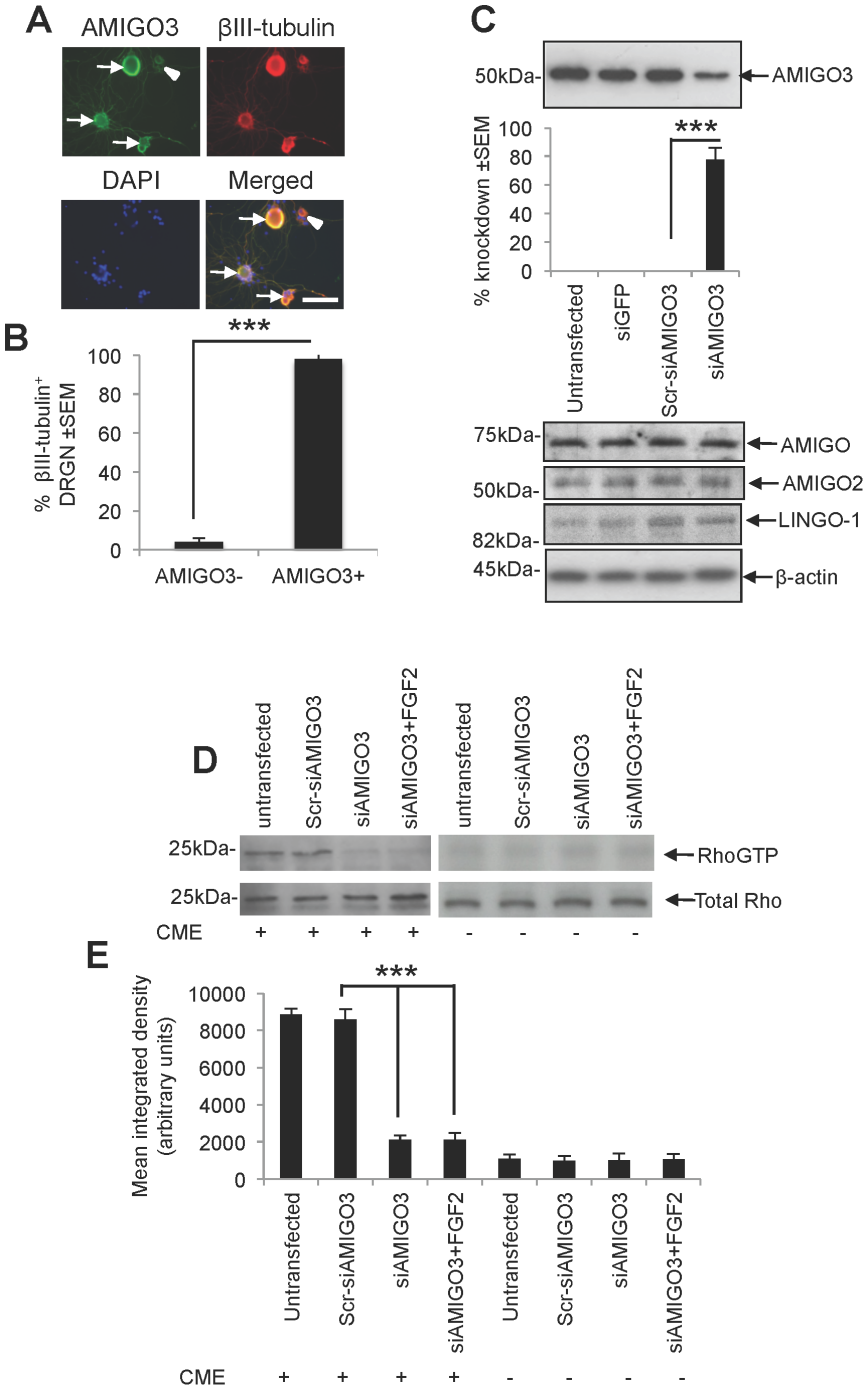
AMIGO3 is localised in DRGN and its suppression using siAMIGO3 in the presence of CME prevents Rho activation. (**A**) Representative AMIGO3 localisation in DRGN (arrows = AMIGO3^+^, arrowhead = AMIGO3_−_) and (**B**) quantification of the percentage ± SEM of DRGN which are AMIGO3^+^ and AMIGO3^−^, in DRG cultures. (**C**) Western blotting and densitometry showing the levels of knockdown, compared to Scr-siAMIGO3 and siGFP after siAMIGO3 treatment. Re-stripped western blots are also shown for AMIGO, AMIGO2 and LINGO-1 to demonstrate that knockdown of AMIGO3 does not affect the levels of these molecules in culture. β-actin is used as a loading control. (**D**) Rho activation assay in untransfected and siRNA transfected DRG cells in the presence of CME. Total Rho levels were also detected by western blotting. Scale bar in (A) = 50 µm.

In dissociated primary adult DRG cultures containing axotomised DRGN, we confirmed immunolocalisation of AMIGO3 protein in >95% of βIII-tubulin^+^ DRGN (**Figure 7A and B**). Transfection of DRG cells with siAMIGO3 knocked down AMIGO3 protein levels by 80% (**Figure 7C**), while no changes in AMIGO3 levels were detected in either untreated cultures or those treated with Scr-siAMIGO3, or siGFP. Transfection of DRG cultures with siAMIGO3 did not affect protein levels of AMIGO, AMIGO2 or LINGO-1, suggesting that the siAMIGO3 was specific to AMIGO3 (**Figure 7C**). In the presence of CME, RhoGTP levels detected by Rho pulldown assay, were high in untransfected DRG cells and after transfection with Scr-siAMIGO3 (**Figure 7D and E**). However, in DRG cells transfected with either siAMIGO3 or siAMIGO3+FGF-2, RhoGTP levels were either low or absent (**Figure 7D and E**). In the absence of CME, untransfected DRG or those transfected with Scr-siAMIGO3, siAMIGO3 or siAMIGO3+FGF2, did not activate Rho (**Figure 7D and E**). These results suggest that knockdown of siAMIGO3 disables the axon growth inhibitory signalling cascade through suppression of RhoA activation that is normally induced by CNS myelin ligands, leading to disinhibition of DRGN neurite outgrowth.

**Figure 8:**
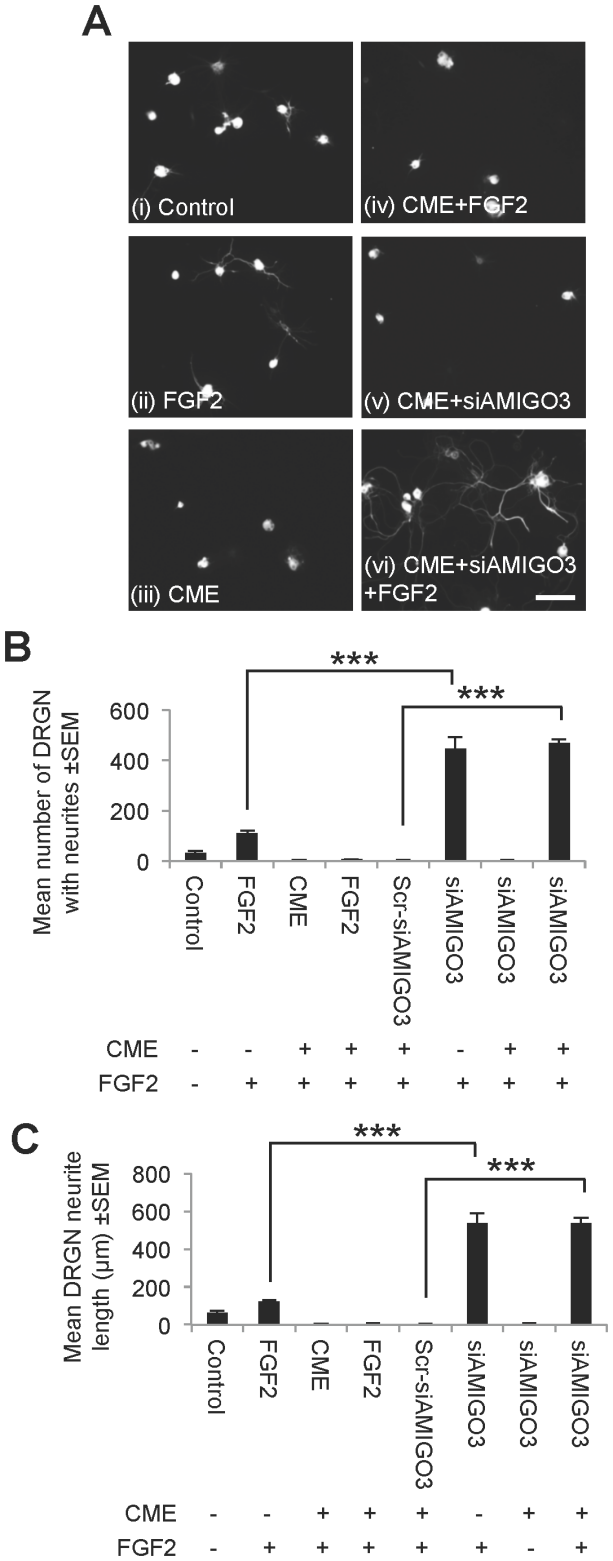
Knockdown of AMIGO3 promotes DRGN neurite outgrowth in the presence of CME. (**A**) Representative photomicrographs of βIII-tubulin^+^ DRGN neurite outgrowth in untransfected and siRNA transfected DRG cells in the presence or absence of CME with or without FGF-2 ± SEM. Quantification of (**B**) mean number of DRGN with neurites ± SEM and (**C**) mean neurite length after treatment with AMIGO3 in the presence of CME, with and without FGF-2 ± SEM. *** = P<0.0001, ANOVA. Scale bar in (A) = 100 µm.

The physiological role of AMIGO3 in blocking neurite outgrowth was confirmed in adult rat DRG cultures. The modest neurite outgrowth seen in untreated DRG cultures (**Figure 8A(i)**) is enhanced by treatment with FGF-2 (**Figure 8A(ii)**), but inhibited by addition of CME (**Figure 8A(iii)**). Neither FGF-2 (**Figure 8A(iv)**) nor siAMIGO3 (**Figure 8A(v)**) treatments alone overcame CME-mediated inhibition. However, the addition of FGF-2 with siAMIGO3 to CME-inhibited cultures promoted vigorous neurite outgrowth (**Figure 8A(vi)**), with enhanced numbers of DRGN (**Figure 8B**) growing longer neurites (**Figure 8C**) compared to untreated and FGF-2-treated DRGN. In the absence of CME, siAMIGO3+FGF2 treatment causes equally robust neurite outgrowth as siAMIGO3+FGF2 in CME-inhibited cultures (**Figure 8B and C**). Taken together, these results demonstrate that siAMIGO3 paralyses the inhibitory NgR1-p75 signalling cascade in the presence of CME and disinhibits DRGN but, despite disinhibition, DRGN neurite outgrowth does not occur unless driven by FGF-2.

### Knockdown of AMIGO3 promotes disinhibited RGC neurite outgrowth in the presence of CME, when stimulated with CNTF

**Figure 9:**
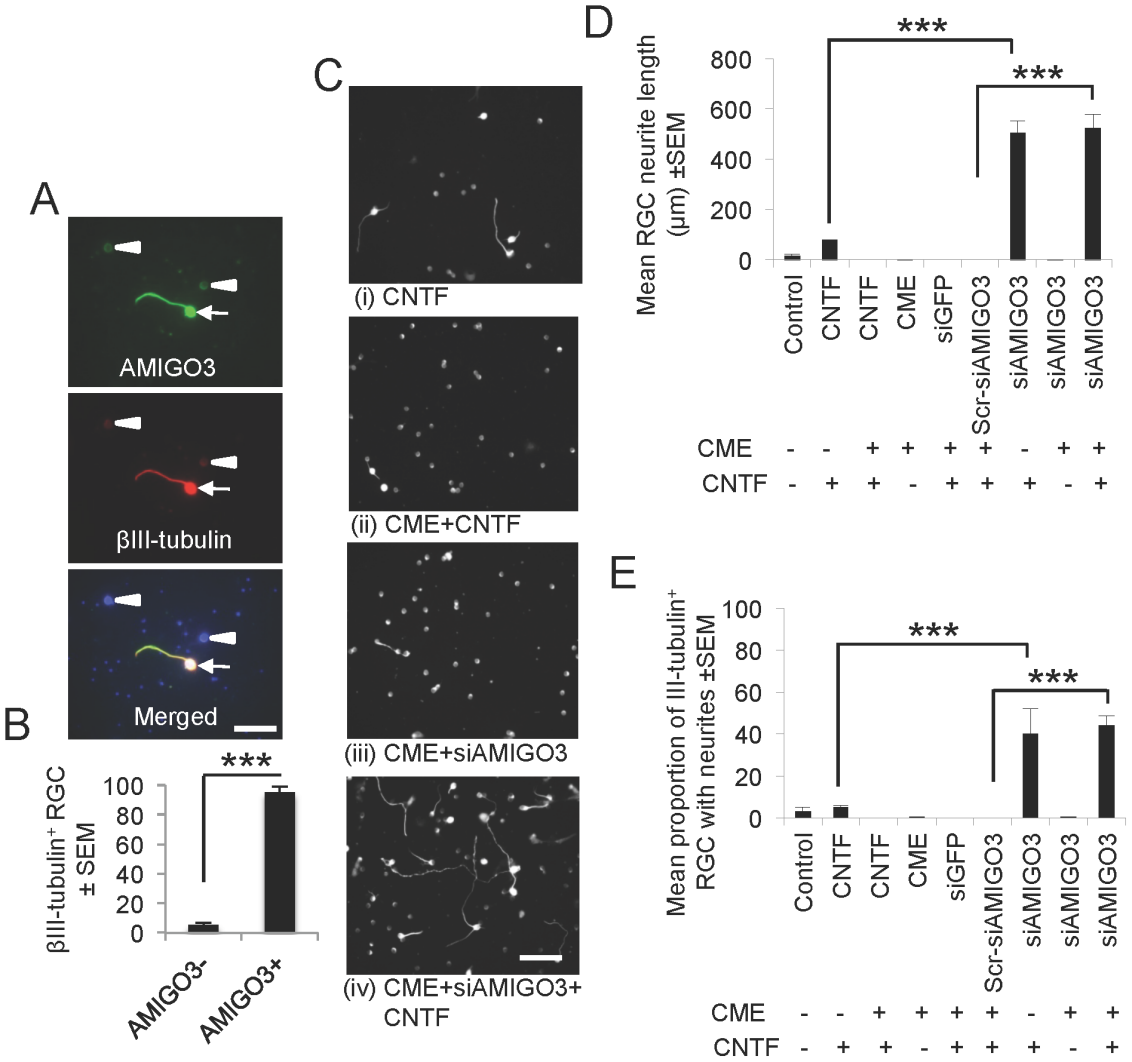
Knockdown of AMIGO3 promote RGC neurite outgrowth in the presence of inhibitory concentration of CME. (A) Immunocytochemistry and (B) quantitation to show that >95% of RGC were AMIGO3^+^ (arrow) while a small proportion of RGC were AMIGO^−^ (arrowheads). (**C**) Representative photomicrographs of βIII-tubulin^+^ RGC neurite outgrowth in CNTF and siRNA transfected retinal cells in the presence and absence of CME with or without CNTF. Quantification of (**D**) mean proportion of RGC with neurites and (**E**) mean neurite length after treatment with siAMIGO3 in the presence of CME, with and without CNTF. *** = P<0.0001, ANOVA. Scale bars in (A) = 50 µm and (C) = 100 µm.

In dissociated adult rat retinal cultures, we first confirmed expression of AMIGO3, before knockdown with the same siAMIGO3 as was used for DRG cultures. We confirmed that >95% of βIII-tubulin^+^ RGC were AMIGO3^+^ (**Figure 9A and B**). The RGC neurite outgrowth observed in CNTF treated retinal cultures (**Figure 9C(i)**) was blocked by the addition of an inhibitory concentration of CME (**Figure 9C(ii)**). However, knockdown of AMIGO3 alone (**Figure 9C(iii)**) did not disinhibit RGC neurite outgrowth in the presence of CME, but stimulation with CNTF after AMIGO3 knockdown promoted significant disinhibited RGC neurite outgrowth in many RGC (**Figure 9C(iv)** **and** **Figure 9D**) which grew longer neurites (**Figure 9E**) compared to untreated or CNTF treated cultures. Knockdown of AMIGO3 in the absence of CME and stimulated with CNTF also produced similar RGC neurite outgrowth as cultures treated with siAMIGO3+CME+FGF2 (**Figure 9D and E**). These results demonstrated that AMIGO3 knockdown in the presence of CME also disinhibits RGC cultures but requires an appropriate neurotrophic factor to drive neurite outgrowth.

## Discussion

Although LINGO-1 is an accepted co-receptor in the NgR1-p75/TROY receptor complex signalling CNS axon growth inhibition, its expression patterns after spinal cord injury are not consistent with its proposed function. Since LINGO-1 levels do not rise appreciably in DRGN until 14 days after injury [10], we postulated that other molecules may take part in signalling axon growth inhibition in the acute phase after injury. In bioinformatic screens from microarray data derived from DRGN of regenerating and non-regenerating DC injury models, we identified AMIGO3 as a possible regeneration-related molecule. Here, we show that AMIGO3 interacts with NgR1, p75 and TROY to constitute a functional receptor complex for CNS myelin-derived inhibitory ligands that activates RhoA and controls axon growth and extension.

We demonstrate a novel role for AMIGO3 as a molecule that participates in the NgR1-p75/TROY receptor complex that signals axon growth inhibition and show that its expression levels are consistent with a physiological function in this complex in the injured CNS. Accordingly, we observed that AMIGO3 mRNA and protein levels were significantly raised in axotomised DRGN in non-regenerating DC lesion models above those seen in regenerating SN and pSN+DC lesioned DRGN from 1 day through to at least 10 days after injury. By contrast, LINGO-1 mRNA and protein remained low acutely and were not modulated between regenerating and non-regenerating paradigms. Since our results showed that AMIGO3 expression levels were significantly higher than those of LINGO-1 in centrally axotomised DRGN and that LINGO-1 was not modulated between regenerating *versus* non-regenerating injury paradigms, we suggest that AMIGO3 may be the preferred receptor partner of the NgR1-p75/TROY axon growth inhibitory signalling complex in the early phase of CNS injury responses. We also confirmed our findings in the DRGN injury model and in our well-established regenerating and non-regenerating ONC injury models [19], [20], [22], [24], [26], where we demonstrated that AMIGO3 levels were elevated in the non-regenerating ON injury model while levels remained low in regenerating ON injury model retinae.

We further show that AMIGO3 interacted with NgR1-p75/TROY in both transfected COS7 cells and in rat and human brain lysates to constitute functional receptor complexes that activated RhoA in response to CNS myelin. This suggests a physiological role of AMIGO3 in mediating myelin-induced axon growth inhibition. The critical role of AMIGO3 in blocking neurite outgrowth was confirmed in both adult rat primary DRG and retinal cultures. Accordingly, siRNA silencing of AMIGO3 paralysed signalling by the NgR1-p75/TROY receptor complex, as evidenced by non-activation of RhoGTP in the presence of CME. Despite this apparent disinhibition of DRGN and RGC, neurite outgrowth did not occur unless driven by an appropriate neurotrophic factor, such as FGF-2 or CNTF. The choice of neurotrophic factor depends on the particular population of neurons under investigation. For example, we have shown that cultured DRG respond optimally to FGF2 [15], [17], [25]; while RGC respond to CNTF [22], [30], [31] and hence these same factors were used in our current study.

The results from both DRG and RGC culture models are consistent with our previous observations that disinhibition alone is not sufficient to promote neurite outgrowth in the presence of CNS myelin but that addition of appropriate neurotrophic factors is required to drive growth. For example, DRGN neurites that were disinhibited by a variety of strategies required either FGF-2 [16], [17], [25], or combinations of nerve growth factor (NGF), brain-derived neurotrophic factor (BDNF) and neurotrophin-3 (NT-3) to elongate neurites [15]. Likewise, RGC neurites that were also disinhibited by a variety of strategies required either combinations of neurotrophic factors such as FGF-2, BDNF and NT-3 [26], [24] or ciliary neurotrophic factor alone to grow neurites [22], [24], [29]. Others have also shown that combining disinhibition with a neurotrophic drive results in enhanced CNS axon regeneration [30], [31], [32].

In summary, our results support a novel role for AMIGO3 as a preferred and mandatory partner in the NgR1-p75/TROY receptor complex that acutely signals the inhibitory effects of CNS myelin-derived ligands. Suppression of AMIGO3 function rather than LINGO-1, when combined with NTF, would be a more effective acute therapeutic strategy to promote CNS axon regeneration.
